# Elucidating Cuproptosis-Associated Genes in the Progression from Nash to HCC Using Bulk and Single-Cell RNA Sequencing Analyses and Experimental Validation

**DOI:** 10.3390/medicina59091639

**Published:** 2023-09-11

**Authors:** Zizuo Zhao, Tiankuo Luan, Jingyuan Wan, Hui Du, Jun Hu, Hao Liu, Xia Gong, Ge Kuang, Bin Wang

**Affiliations:** 1 Department of Anesthesiology, The First Affiliated Hospital of Chongqing Medical University, Chongqing 400016, China; zhaozz513@hospital.cqmu.edu.cn; 2 Department of Anatomy, Chongqing Medical University, Chongqing 400016, China; 2020110055@stu.cqmu.edu.cn (T.L.); xiagong@cqmu.edu.cn (X.G.); 3 Department of Pharmacology, Chongqing Medical University, Chongqing 400016, China; jywan@cqmu.edu.cn (J.W.); duhui_jam@163.com (H.D.); hjun952026@126.com (J.H.); 2020111579@stu.cqmu.edu.cn (H.L.)

**Keywords:** FDX1, NASH to HCC, immune landscape, cuproptosis

## Abstract

*Background and Objectives*: Non-alcoholic steatohepatitis (NASH) is a significant risk factor for hepatocellular carcinoma (HCC) development. Timely treatment during the NASH stage is essential to minimize the possibility of disease progression to HCC. Cuproptosis is a newly identified form of cellular death that could impact the progression of various diseases and cancers. *Materials and Methods*: Transcriptome and single-cell sequencing datasets were utilized to investigate the role of cuproptosis-related genes (CRGs) in NASH progression to HCC. FDX1, LIPT1, and PDHP were identified as CRGs in NASH patients, and FDX1, DBT, GCSH, SLC31A1, and DLAT were identified as CRGs in patients with NASH progressing to HCC. FDX1 was found to play a significant role in both NASH patients and patients with NASH progressing to HCC. This study constructed cuproptosis-related clusters (CRCs) using the Nonnegative Matrix Factorization algorithm, and they were linked to fatty acid metabolism and the PPAR signaling pathway in both NASH CRCs and HCC CRCs. The Weighted Correlation Network Analysis algorithm identified CRP, CRC, TAT, CXCL10, and ACTA1 as highly relevant genes in NASH CRCs and HCC CRCs. The expression of FDX1 was validated in both mouse models and human NASH samples. *Results*: The investigation highlights FDX1 as a pivotal CRG in both NASH and NASH progression to HCC. The comprehensive characterization of CRGs sheds light on their potential biofunctional importance in the context of NASH and HCC. Our experimental results show that FDX1 expression was significantly increased in NASH patients. *Conclusions*: The present study identified key CRGs, revealing their potential impact on NASH and HCC. Meanwhile, targeting FDX1 may prevent the progression of NASH to HCC.

## 1. Introduction

The liver serves as the primary metabolic organ within the human body and plays a crucial integrative role in the metabolism of glycogen, protein, and fat. Non-alcoholic fatty liver disease (NAFLD) is a prevalent chronic liver disorder that is prevalent in both developing and developed countries [1]. Its pathogenesis is primarily attributed to an excessive energy intake coupled with an inadequate lifestyle, which leads to the accumulation of triglycerides within the liver, resulting in cellular damage, inflammation, and dysfunction. A subset of NAFLD, NASH, characterized by the presence of inflammatory cells and liver cell injury, can progress to more severe stages of liver fibrosis, cirrhosis, and even HCC if left untreated [2,3]. Thus, early diagnosis and effective management are crucial for preventing the progression of this debilitating and potentially fatal disease [4]. NASH is a form of non-alcoholic fatty liver disease characterized by the presence of liver cell injury and inflammation [5]. This condition is considered a primary cause of HCC, a malignant tumor that affects the liver. Given the high incidence of NASH and HCC, early prevention of the development of NASH is of paramount importance for patients. Despite the significant clinical need, the currently available treatments for NASH are inadequate, and the identification of effective therapies remains an ongoing area of research. Therefore, the early detection of NASH biomarkers and identification of therapeutic targets is crucial in preventing the progression of this disease to HCC and improving the overall survival rate [6]. This highlights the importance of basic and translational research efforts aimed at understanding the underlying mechanisms and developing novel interventions for the management of NASH. The discovery and validation of specific biomarkers and drug targets that are specific to NASH may serve as key tools for early diagnosis and treatment of the disease, thereby reducing the risk of HCC and enhancing the overall prognosis for affected individuals. Simultaneously, endeavors have been made to prognosticate efficacious biomarkers that can offer assistance to second-line immunotherapy [7].

Recently, Tsvetkov et al. conducted a study in which they investigated the effects of copper ion carriers with distinct structural characteristics on 489 diverse cell lines [8]. Their results demonstrate that copper ion carriers can induce cell death, a form of programmed cell death known as cuproptosis. Cuproptosis involves the excessive accumulation of copper ions, resulting in the anomalous aggregation of thioctylated proteins, disrupting iron–sulfur cluster proteins associated with mitochondrial respiration, and eliciting a proteotoxic stress response, ultimately culminating in cell demise. This study adds to a growing body of literature indicating that targeting copper metabolism may be a viable strategy for enhancing the sensitivity of cancer cells to chemotherapy. This highlights the potential therapeutic value of manipulating copper homeostasis in the treatment of cancer.

Cuproptosis is a recently identified form of programmed cell death that diverges from established mechanisms, such as apoptosis, pyroptosis, necrosis, and ferroptosis [9]. It is characterized by the pathological accumulation of copper within a cell, which surpasses the ability of cellular homeostatic mechanisms to control it [10]. The binding of copper ions to acylated components of the tricarboxylic acid cycle leads to the aggregation of acylated proteins and the depletion of iron–sulfur cluster proteins, thereby inducing protein toxicity and ultimately culminating in cellular demise.

Recent studies have indicated that CRGs may serve as prognostic indicators for various types of cancer, such as clear-cell renal-cell carcinoma, neck squamous cell carcinoma, pancreatic cancer, and breast cancer [11]. Additionally, these genes have been found to be strongly associated with the immune microenvironment. Copper depletion therapy has been shown to effectively inhibit the migration of cancer cells in patients with metastatic breast cancer [12]. Furthermore, combination therapy of copper chelators with BH3 mimetic agents has demonstrated efficacy in treating a specific type of melanoma caused by a V600E mutation in the BRAF gene [13].

In the case of HCC, a study has identified five cuproptosis-mediated pattern-related genes, namely, PBK, mMP1, gNAZ, GPC1, and AKR1D1, that were effective in evaluating the prognosis of HCC patients [14]. However, research on the relationship between NASH progression to HCC and CRGs is still limited. Therefore, further studies should be conducted to explore the association between NASH, NASH progression to HCC, and CRGs.

In this study, based on the expression of CRGs, we used a variety of bioinformatics algorithms to divide NASH patients and patients who progressed from NASH to HCC into two cuproptosis-related pattern subtypes. The potential biological function and immune cell activity changes and CRGs in the different subtypes were comprehensively analyzed.

## 2. Materials and Methods

### 2.1. Data Collection

The GSE164760 expression matrix was downloaded from GEO (https://www.ncbi.nlm.nih.gov/geo/, accessed on 20 August 2023). The dataset consists of a total of 170 samples, including healthy livers, cirrhotic livers (*n* = 8), NASH livers (*n* = 74), NASH-HCC adjacent non-tumor tissues (*n* = 29), and NASH-HCC tissues (*n* = 53). Healthy livers (*n* = 6), NASH patients (*n* = 74), and patients with NASH progressing to HCC (*n* = 53) were extracted from GSE164760 for subsequent analysis. Eleven CRGs (FDX1, LIPT1, LIAS, DLD, DBT, GCSH, DLST, DLAT, PDHA1, PDHB, and SLC31A1) were collected from the literature (Supplementary Table S1).

### 2.2. Gene Set Enrichment Analysis (GSEA) and OS Analysis of CRGs

An online survival analysis tool (Kaplan–Meier plotter (kmplot.com)) was used to analyze the effects of FDX1, DBT, DLAT, SLC31A1, and GCSH in patients with liver cancer [15]. The impact of FDX1 from NASH to HCC patients was analyzed using GSEA [16].

### 2.3. Construction of Nomogram Model for CRGs

A nomogram model of copper-death-related genes was constructed using the R package “rms”. We scored each gene based on the degree of contribution of cuproptosis-related genes to the outcome variable and then summed the individual gene scores to obtain the total score. The total score was used to evaluate NASH patients and patients with NASH progressing to HCC.

### 2.4. Construction and Verification of CRC for NASH and NASH Development to HCC

The determination of CRC was based on the algorithm of the R package “ConsensusClusterPlus” for a consistency clustering analysis [17]. Consistent clustering verifies the clustering rationality by using a resampling-based approach, whose main purpose is to assess the stability of the clusters. The “pROC” package was used to plot ROC curves to determine the diagnostic value of cuproptosis-related genes for the disease [18].

### 2.5. Assessment of Immune Cell Infiltration CRC Using ssGSEA

The ssGSEA algorithm was used to calculate the difference in 23 immune cell infiltrations between cuproptosis-related clusters. Three R packages, namely, “Limma”, “GSEABase”, and “GSVA”, were applied to complete the analysis [19].

### 2.6. Data Processing and Analysis of Single Cells

We used the (https://tabula-muris.ds.czbiohub.org/, accessed on 30 August 2023) website selection fluorescence-activated cell sorting (FASC) method to analyze the association between FDX1 gene expression levels and multiple immune cells in mouse liver. To investigate the differences in the immune cell expression of FDX1 in healthy and NASH livers, we performed data merging and quality control on samples from GSE210501 using the “Seura” package and “SingleR” package [20]. The quality control conditions were as follows: cells were excluded if they expressed less than 50 genes per cell and more than 5% mitochondrial gene expression.

### 2.7. Weighted Gene Co-Expression Network Analysis (WGCNA)

We used the WGCNA algorithm to analyze the gene expression patterns of cuproptosis-related gene clusters, clustered genes with similar expression patterns, and analyzed the association between modules and specific traits or phenotypes. An adjacency matrix was constructed using a weighted-phase relationship method. The WGCNA algorithm completes the analysis in four steps: the calculation of correlation coefficients between genes and the identification of gene modules, co-expression networks, and module–trait associations [21]. The cuproptosis characteristic cluster module genes were finally identified for subsequent analysis. The R packages “WGCNA” and “limma” were used to implement the WGCNA analysis.

### 2.8. Human Tissue Specimens

Human NASH tissues (*n* = 4) and normal samples adjacent to liver cancer (*n* = 4) were used for an immunohistochemistry (IHC) staining analysis; samples were provided by the First Affiliated Hospital of Chongqing Medical University (Chongqing, China).

### 2.9. Reagents, Animals, and Animal Experiments

Kits produced by Nanjing Jiancheng Institute of Biological Engineering (Nanjing, China) were used to detect alanine aminotransferase (ALT), aspartate aminotransferase (AST), and triglycerides (TGs). An anti-FDX1 antibody was purchased from “Bioword” (Nanjing, China). Male C57BL/6J mice aged 6–8 weeks (20–25 g) were purchased from the Animal Center of Chongqing Medical University. The animals used for the experiments had adequate food and water and were housed in a stable environment at 20–25 °C with a circadian rhythm and humidity maintained at 55 ± 5%. We gave the animals at least one week to acclimatize before conducting experiments. The animal experiments covered in this article were carried out in strict accordance with the guidelines of the Chongqing Medical University Animal Care and Use Committee. We divided the mice randomly into two groups (*n* = 5): a normal diet (ND) group and a Western-diet (WD) group. The ND group was fed a standard chow diet, and the WD group was fed a Western diet for 16 weeks to induce the occurrence of NASH. The diets of the mice mentioned above were purchased from Trophic Animal Feed High-tech Co. Ltd. (Jiangsu, China). After 16 weeks, we sacrificed the mice with sevoflurane. Blood was collected from the retro-orbital sinus, and liver tissue was collected from the mice for subsequent analysis.

### 2.10. Biochemical Analysis

The liver samples from the mice were churned through a tissue homogenizer and centrifuged, and supernatant was obtained. The blood samples from the mice were centrifuged to obtain serum.

ALT, AST activity in mouse serum, and TG content in the liver were determined according to the instructions in the kit from the Nanjing Jiancheng Bioengineering Institute (Nanjing, China).

### 2.11. Immunohistochemistry

The protocol of the IHC staining was based on a previous article published by our laboratory [22]. The IHC staining of the mice and human liver samples was carried out using anti-FDX1 (1:100).

### 2.12. Oil Red O Staining

The mice’s liver tissues were preserved in an optimum cutting temperature (OCT) compound, cut to a thickness of 15 μm, and placed on slides, followed by fixation in 75% alcohol. Next, the frozen sections were stained using an Oil Red O solution, and the nuclear section was stained with hematoxylin as a counterstain.

### 2.13. Histology Examination

The liver tissue was formalin-fixed, dehydrated, embedded in paraffin, and finally prepared into 5 μm sections. The H&E staining method was applied to examine the liver tissues.

### 2.14. Statistics

In this study, all expression matrix data were analyzed using R software v4.1.2. The Wilcoxon test was used to compare the differences in the mRNA expression of CRGs between the two groups. The results of the animal experiments were analyzed using two-tailed Student’s t-test for the ND and WD groups, and a one-way ANOVA was performed for multiple comparisons (*p* < 0.05 = “*”, *p* < 0.01 = “**”, *p* < 0.001 = “***”, *p* > 0.05 = ns). *p* values < 0.05 indicated statistical significance.

## 3. Results

### 3.1. Identification of Key CRGs in the Transition of NASH to HCC

**Figure 1:**
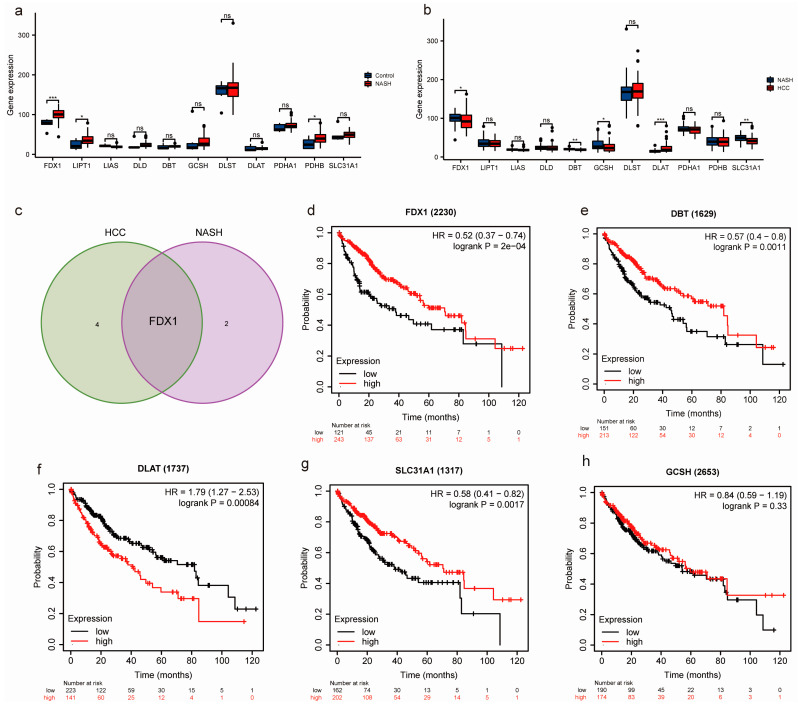
Identification of key CRGs in NASH and NASH-induced HCC. (**a**,**b**) Box plot showing the expression profiles of key CRGs. *p* < 0.05 = *, *p* < 0.01 = **, *p* < 0.001 = ***. No statistical significance is denoted by ns. (**c**) Venn diagrams were employed to determine the overlap between critical CRGs linked to NASH and crucial CRGs involved in NASH progression to HCC. (**d**–**h**) Assessment of the prognostic value of FDX1, DBT, GCSH, SLC31A1, and DLAT.

To investigate whether there are differences in the expression of CRGs during the progression from NASH to HCC, we extracted the mRNA expression matrix of 11 CRGs in GSE164760. We utilized box plots to visually depict the variation in CRGs between healthy livers and livers affected by NASH (Figure 1a) and, similarly, the variation in CRGs between NASH-affected livers and HCC tissues that progressed from NASH to HCC (Figure 1b). FDX1, LIPT1, and PDHP were significantly upregulated in the NASH patients. FDX1, DBT, GCSH, and SLC31A1 were obviously lower in the HCC patients, but DLAT expression was significantly higher. The expression of FDX1 varied significantly from NASH to HCC (Figure 1c). Moreover, we used the Kaplan–Meier plotter tool to analyze the overall survival of patients with liver cancer based on the expression of the FDX1, DBT, GCSH, SLC31A1, and DLAT genes. FDX1, DBT, and SLC31A1 expression levels were significantly associated with good over survival (OS) in patients. In contrast, DALT expression was associated with poor OS, and GSCH expression was not significantly associated with OS (Figure 1d–h).

### 3.2. Analysis of the Immune Microenvironment of NASH to HCC

**Figure 2:**
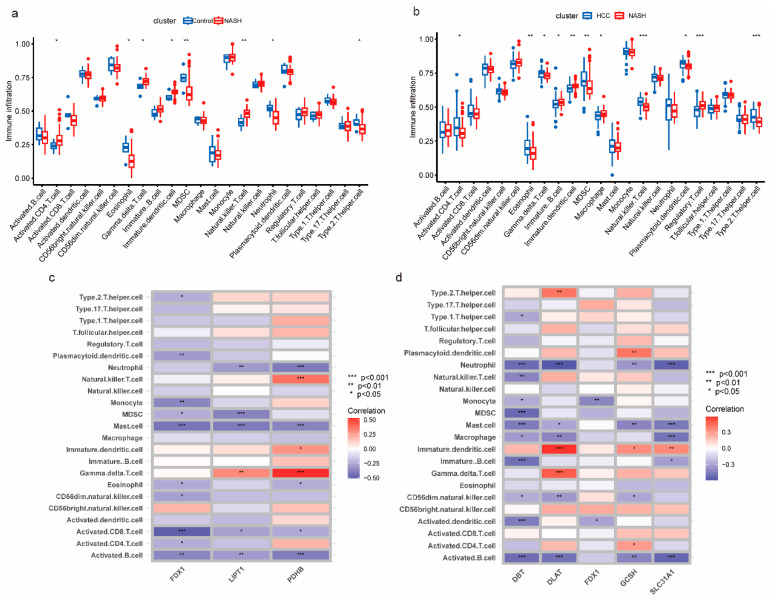
Landscape of immune infiltration in NASH patients and NASH to HCC patients. (**a**,**b**) Box plot presenting the proportion of immune cell infiltration in patients at different stages. (**c**,**d**) Heat map of the correlation between key CRGs and immune cells. *p* < 0.05 = *, *p* < 0.01 = **, *p* < 0.001 = ***.

Later, we compared the immune cell infiltration in patients in the control and NASH groups, as well as in NASH patients and NASH to HCC patients. The results in Figure 2a and b show that activated CD4 T cells, gamma delta T cells, immature B cells, immature DC cells, and natural killer T cells were consistently elevated and significantly different in both the NASH and NASH to HCC groups. Within the continuum from NASH to HCC progression, a predominant fraction of key CRGs displayed a negative association with activated B cells, CD56dim natural killer cells, mast cells, and neutrophils while manifesting a positive connection with gamma delta T cells and immature dendritic cells (Figure 2c,d).

### 3.3. Construction of Nomogram Models Based on the Development of NASH to HCC

**Figure 3:**
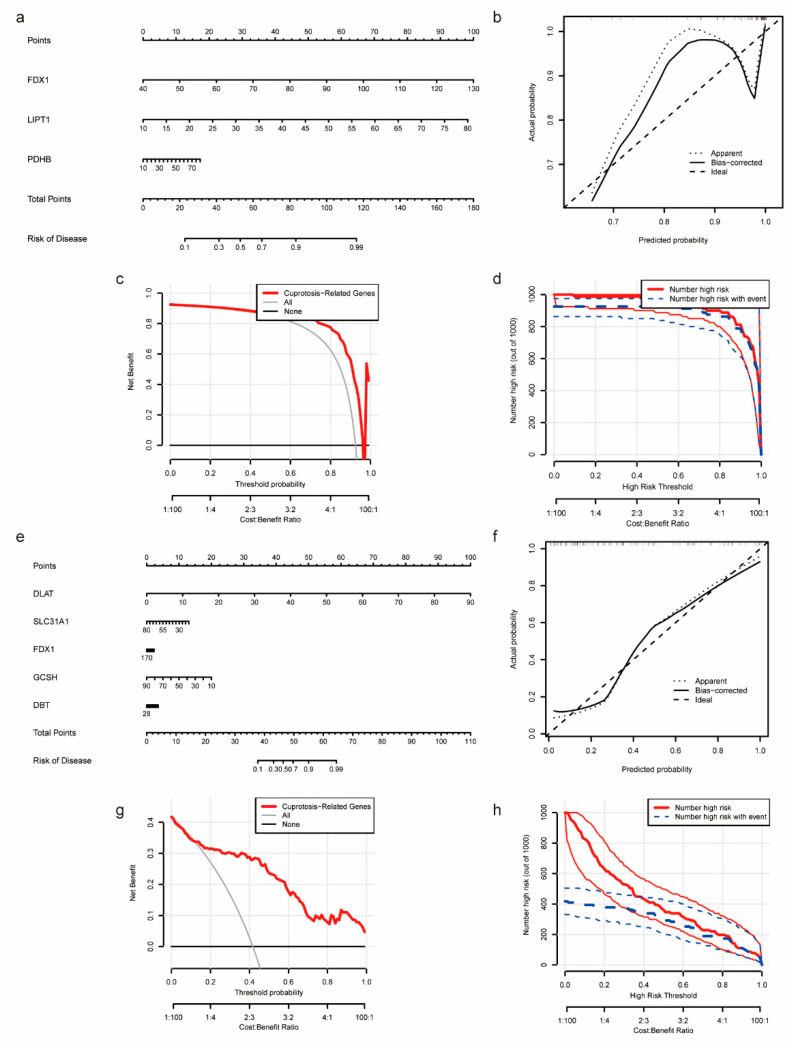
Construction of nomogram of NASH and NASH-induced HCC to assess clinical value. (**a**) Nomogram demonstrates the prognostic value of FDX1, LIPT1, and PDHP for NASH patients. (**b**) Calibration curves to assess the degree of similarity between the predicted and true results of the NASH nomogram. (**c**) Decision Curve Analysis to evaluate the sensitivity and specificity of NASH nomogram. (**d**) Clinical impact curve to assess the clinical impact of the NASH nomogram at different thresholds. (**e**) Nomogram demonstrates the prognostic value of FDX1, DBT, GCSH, SLC31A1, and DLAT for NASH-induced HCC patients. (**f**) Calibration curves to assess the degree of similarity between the predicted and true results of the NASH-induced HCC nomogram. (**g**) Decision Curve Analysis to evaluate the sensitivity and specificity of the NASH-induced HCC nomogram. (**h**) Clinical impact curve to assess the clinical impact of the NASH-induced HCC nomogram.

In order to provide targeted diagnostic and therapeutic advice to patients suffering from NASH or HCC, we developed nomogram models for the NASH patients (utilizing FDX1, LIPT1, and PDHP expression) and the NASH to HCC patients (utilizing FDX1, DBT, GCSH, SLC31A1, and DLAT expression) (Figure 3a,e). As evidenced by the calibration curves, the nomogram model based on CRGs showed a high degree of prediction accuracy and similarity with the actual positive rate (Figure 3b,f)). A Decision Curve Analysis (DAC) and a clinical impact curve analysis (CICA) showed that the nomogram model constructed from the copper-death-related genes is important for patients who have progressed from NASH to HCC (Figure 3c,d,g,h).

### 3.4. Construction, Validation, and Biological Characterization of a Cuproptosis-Related Cluster

**Figure 4:**
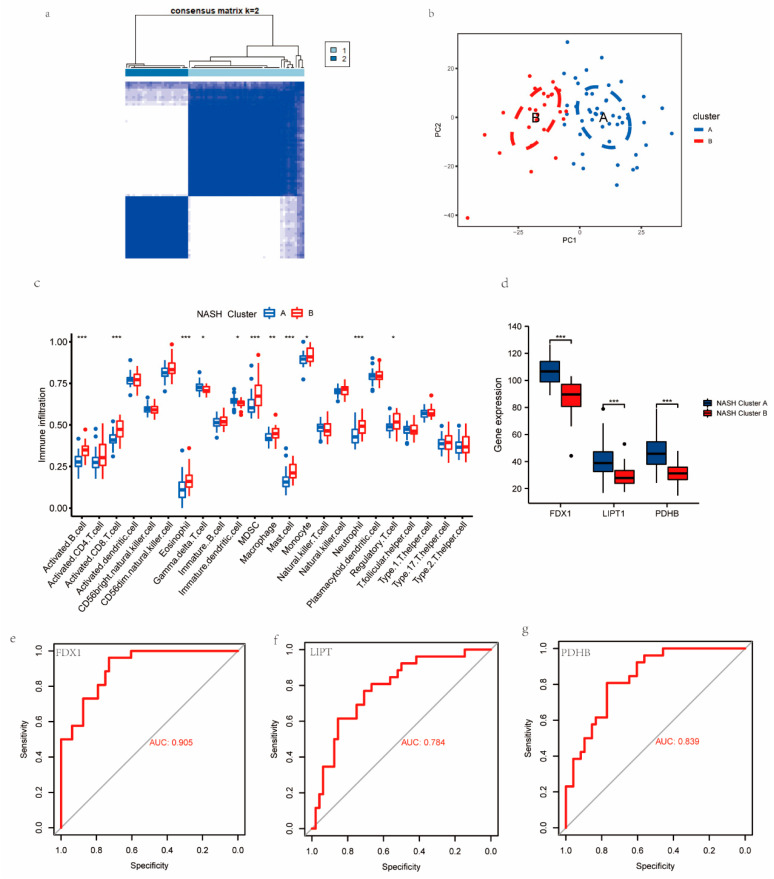
Establishing NASH CRCs based on key CRGs. (**a**) Two NASH CRCs were established using the NFM algorithm, consensus matrix (k = 2). (**b**) PAC analysis of NASH Cluster A and NASH Cluster B. (**c**) Infiltration of 23 immune cells in NASH Cluster A and NASH Cluster B. (**d**) Expression of FDX1, LIPT1, and PDHB in NASH Cluster A and NASH Cluster B. *p* < 0.05 = *, *p* < 0.01 = **, *p* < 0.001 = ***. (**e**–**g**) ROC analysis results on FDX1, LIPT1, and PDHB.

**Figure 5:**
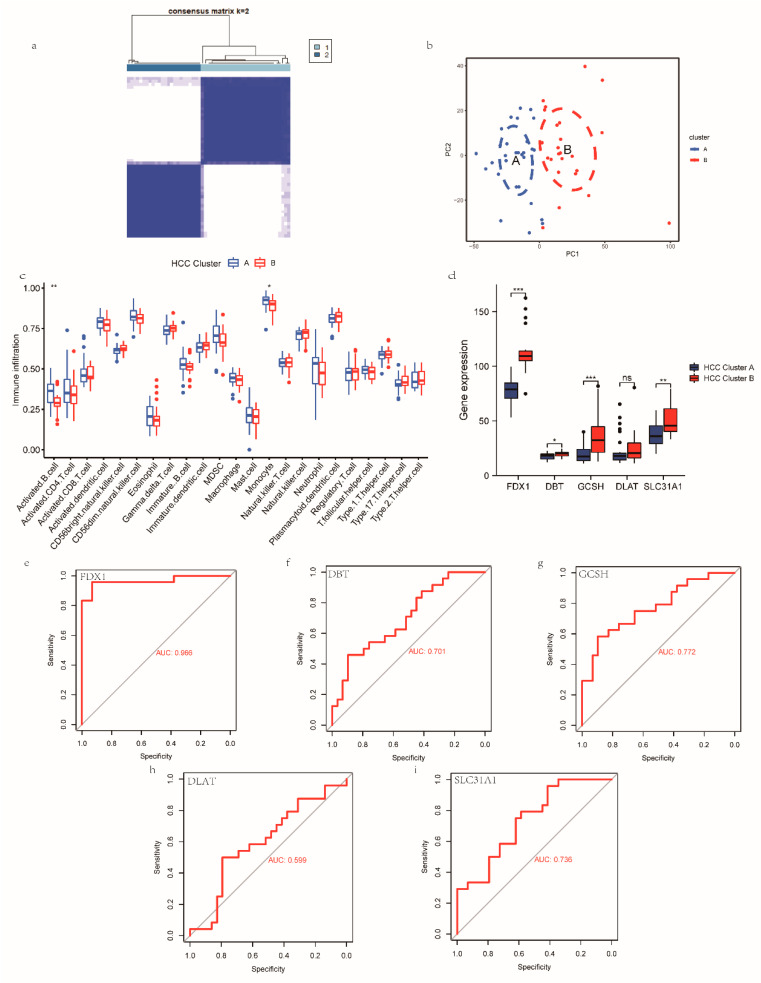
Establishing HCC CRCs based on key CRGs. (**a**) Two HCC CRCs were established using the NFM algorithm, consensus matrix (k = 2). (**b**) PAC analysis of HCC Cluster A and HCC Cluster B. (**c**) Infiltration of 23 immune cells in HCC Cluster A and HCC Cluster B. (**d**) Expression of FDX1, DBT, GCSH, DLAT, and SLC31A1 in HCC Cluster A and HCC Cluster B. *p* < 0.05 = *, *p* < 0.01 = **, *p* < 0.001 = ***. No statistical significance is denoted by ns. (**e**–**i**) ROC analysis results on FDX1, DBT, GCSH, DLAT, and SLC31A1.

We aimed to investigate more profoundly the underlying role of CRGs in the progression of NASH to HCC. Based on the expression of the FDX1, LIPT1, and PDHP genes in the NASH patients, we classified the NASH patients into two clusters: NASH cuproptosis-related clusters (NASH CRCs) A and B. For NASH CRC A, 48 samples were analyzed, and 26 samples were analyzed for NASH CRC B (Figure 4a). The same algorithm was adopted to classify the HCC patients into HCC cuproptosis-related clusters (HCC CRCs) A and B based on the expression of the FDX1, DBT, GCSH, SLC31A1, and DLAT genes in the HCC patients who developed NASH, with 29 samples in HCC CRC A and 24 samples in HCC CRC B (Figure 5a). PCA analysis demonstrated that both NASH CRCs and HASH CRCs could be well partitioned using CRGs (Figure 4b and Figure 5b). In the following step, we analyzed the infiltration of 23 immune cells in both NASH CRCs and HCC CRCs separately using the ssGSEA algorithm. B cells, T cells, mast cells, monocytes, neutrophils, and regulatory T cells were detected in high abundance in NASH CRC B. NASH CRC A infiltration was significantly characterized by gamma delta T cells and immature dendritic cells (Figure 4c). In HCC CRCs, the infiltration proportion of activated B cells and monocytes in HCC CRC A was significantly higher than that in HCC CRC B (Figure 5c). In NASH CRC A and NASH CRC B, FDX1, LIPT1, and PDHP were significantly different, while FDX1, DBT, GCSH, and SLC31A1 were significantly different in HCC CRC A and HCC CRC B (Figure 4d and Figure 5d). A good classification function can be observed in the ROC curves of FDX1, LIPT1, and PDHP in NASH CRCs (Figure 4e–g), as well as in those of FDX1, DBT, GCSH, DALT, and SLC31A1 in HCC CRCs (Figure 5e–i). These findings provide additional validation to support the feasibility of our model concerning cuproptosis-associated traits in individuals progressing from NASH to HCC.

### 3.5. Functional Analyses Based on the NASH CRC to HCC CRC and PPI Construction

**Figure 6:**
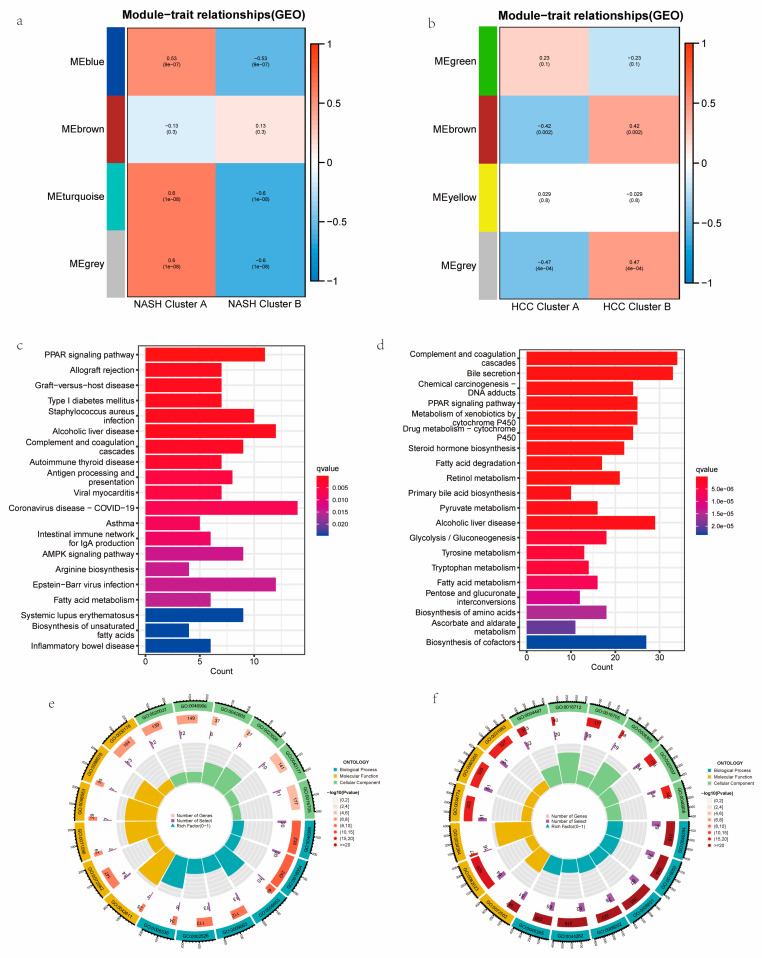
Functional enrichment analysis of core module genes of co-expression network between NASH CRCs and HCC CRCs. (**a**) Selection of core modules in NASH CRCs. (**b**) Selection of core modules in HCC CRCs. (**c**) KEGG analysis of the NASH CRC gray module. (**d**) KEGG analysis of the HCC CRC gray module. (**e**) GO analysis of the NASH CRC gray module. (**f**) GO analysis of the HCC CRC gray module.

**Figure 7:**
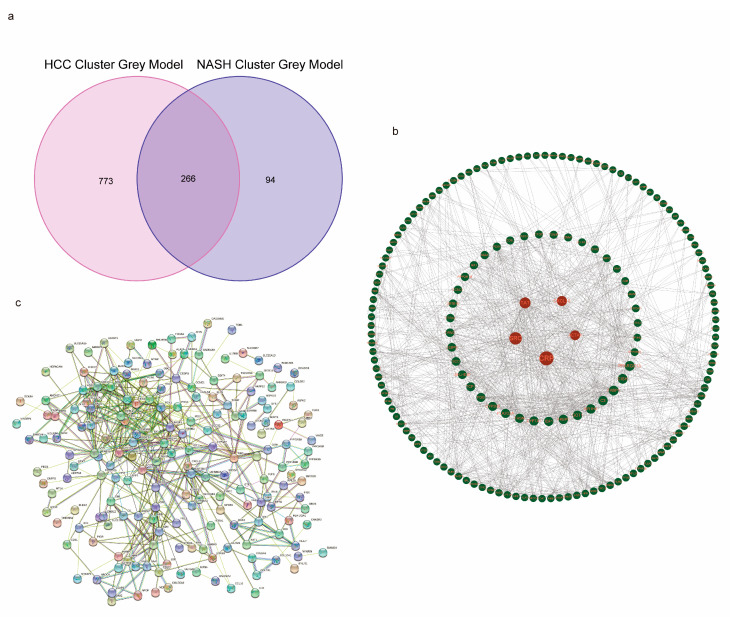
PPI analysis of overlap genes in NASH CRCs and HCC CRCs. (**a**) NASH CRC gray module and HCC CRC gray module overlapping genes. (**b**) PPI network diagram of overlapping genes. (**c**) The top 5 hub genes were calculated using betweenness centrality algorithm. The genes in the red circles have high correlation.

The above results further validate the plausibility of our model concerning cuproptosis-related characteristics in patients with NASH progressing to HCC. In order to analyze NASH CRCs and HCC CRCs separately, we used the WGCNA algorithm and chose the module that had the greatest significance *p*-value and the highest correlation for use in the subsequent analysis (Figure 6a,b). For the KEGG and GO enrichment analyses of NASH CRCs and HCC CRCs, we extracted 360 genes from the gray module of NASH CRCs and 1039 genes from the gray module of HCC CRCs, respectively. The KEGG results showed that both the NASH CRC gray module and the HCC CRC gray module were tightly linked to the PPAR signaling pathway and fatty acid metabolism (Figure 6c,d and Supplementary Table S2). At the gene ontology level, both the NASH CRC gray module and the HCC CRC gray module enrichment results were relevant to organic acid biosynthetic processes, organic acid catabolic processes, fatty acid metabolic processes, blood microparticles, and redox enzyme activity (Figure 6e,f and Supplementary Table S3). As a further step, we intersected the NASH CRC gray module with the HCC CRC gray module in order to obtain 266 key genes (Figure 7a) and mapped the PPI network using the STRING tool (Figure 7c). We used the betweenness centrality algorithm to identify genes strongly associated with cuproptosis in the patients who progressed from NASH to HCC, resulting in TOP5 genes (CRP, CRC, TAT, CXCL10, and ACTA1) being core genes (Figure 7b); details are shown in Supplementary Table S4.

### 3.6. Single-Cell Analysis of Healthy Mouse Hepatocytes and Mouse NASH Hepatocytes

**Figure 8:**
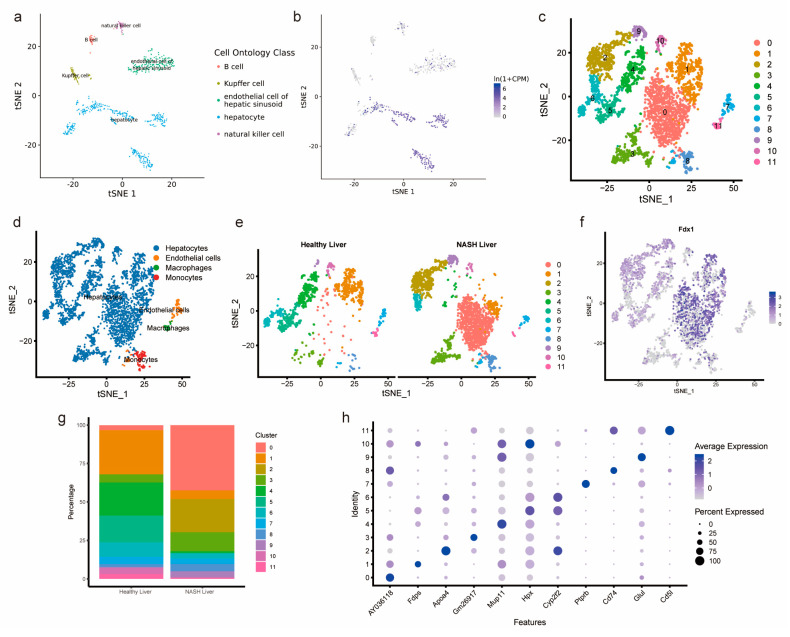
Analysis of Fdx1 expression in mouse immune cells using single-cell analysis. (**a**,**b**) Tabula Muris tool displaying the expression of Fdx1 in different immune cells of mice. (**c**) t-SNE plots showing multiple cell subpopulations. (**d**) Specific notes of 11 cell clusters. (**e**) t-SNE plots comparing immune cell heterogeneity in healthy liver and NASH liver. (**f**) t-SNE plots showing the expression of FDX1 on immune cells. (**g**) Proportional differences of 11 cell clusters. (**h**) Dot plot showing the most representative genes in the 11 cell clusters.

Interestingly, we found that, in patients with NASH-induced HCC, FDX1 displays significant performance. (Figure 1c). The results of the Tabula Muris tool analysis showed that Fdx1 was mainly expressed in mouse hepatocytes (Figure 8a,b). Using GSE210501, we investigated the heterogeneity between healthy mouse livers and livers with NASH. We used the R package “Seurat package” to finally calculate 11 cell clusters and used “single R” to compare mouse RNA seq data for cell–bead cell subpopulation annotation to finally obtain 4 cell subpopulations of hepatocytes, endothelial cells, macrophages, and monocytes (Figure 8c,d). In NASH liver, Cluster 0 cells were mostly concentrated, whereas only a small number of Cluster 0 cells were present in healthy liver (Figure 8e). Fdx1 was mainly expressed in hepatocytes and was most intensively expressed in Cluster 0 (Figure 8f). The cell proportions of the healthy liver and NASH liver and the TOP genes of the 11 cell clusters are shown in Figure 8g and Figure 8h, respectively.

### 3.7. Validation of FDX1 Expression in NASH Models and Human NASH Samples

**Figure 9:**
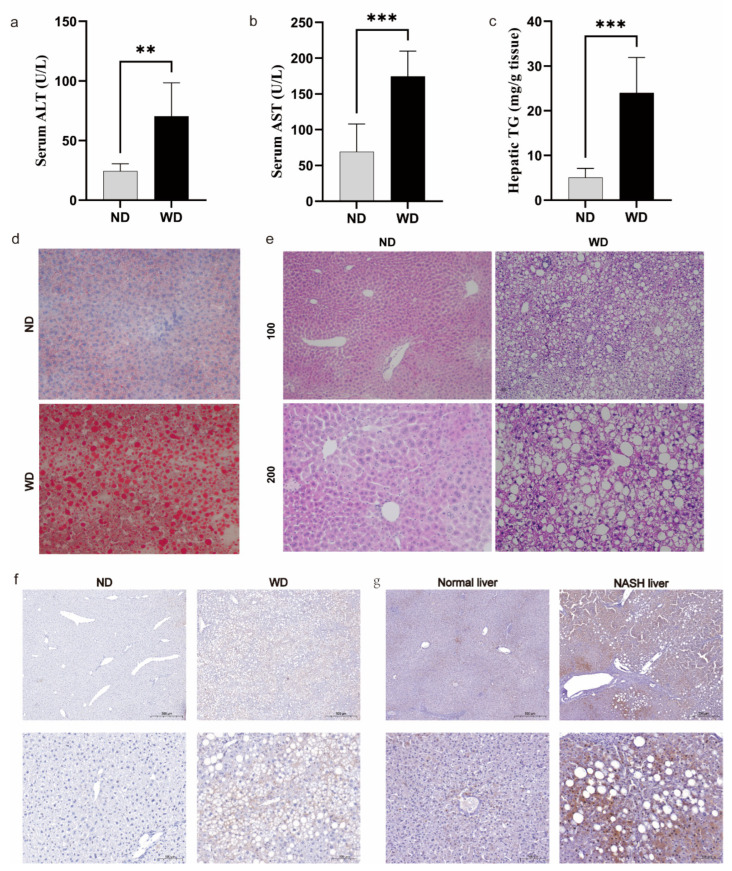
Expression of Fdx1 in WD mouse models and human NASH samples. (**a**) Determination of ALT in serum. (**b**) Determination of AST in serum. (**c**) TG content in mice liver samples. (**d**) Detection of lipid droplets in mice liver samples using Oil Red O staining (200×). (**e**) Presentation of liver pathology results using HE staining. (**f**) Fdx1 expression in mice liver samples using immunohistochemical methods. Scale bars: 100 μm and 500 μm. (**g**) FDX1 expression in normal tissues adjacent to HCC and NASH tissues using immunohistochemical methods. Scale bars: 100 μm and 500 μm. *p* < 0.01 = **, *p* < 0.001 = ***.

To explore FDX1′s impact on NASH development, we established a NASH model using a Western diet. We then gauged serum ALT and AST activity, alongside liver TG content, in both the ND and WD groups. There was a significant difference between the WD and ND groups in terms of the levels of ALT, AST, and TG activities (Figure 9a–c). Lipid droplets in the WD group were much larger and more numerous than those in the ND group (Figure 9d). The ND group had no liver lesions, whereas the WD group had hepatic steatosis, lobular inflammatory lesions, and ballooning of hepatocytes (Figure 9e). The above experimental results sufficiently illustrate the reliability of our NASH model construction. FDX1 was widely expressed around hepatocyte steatosis, as shown in the IHC results; this result was consistent in both the mouse NASH samples and human NASH samples (Figure 9f,g). In conclusion, the experimental results are consistent with our bioinformatics analysis, suggesting that FDX1 may affect the course of NASH.

### 3.8. GSEA Evaluation Based on FDX1 Expression

**Figure 10:**
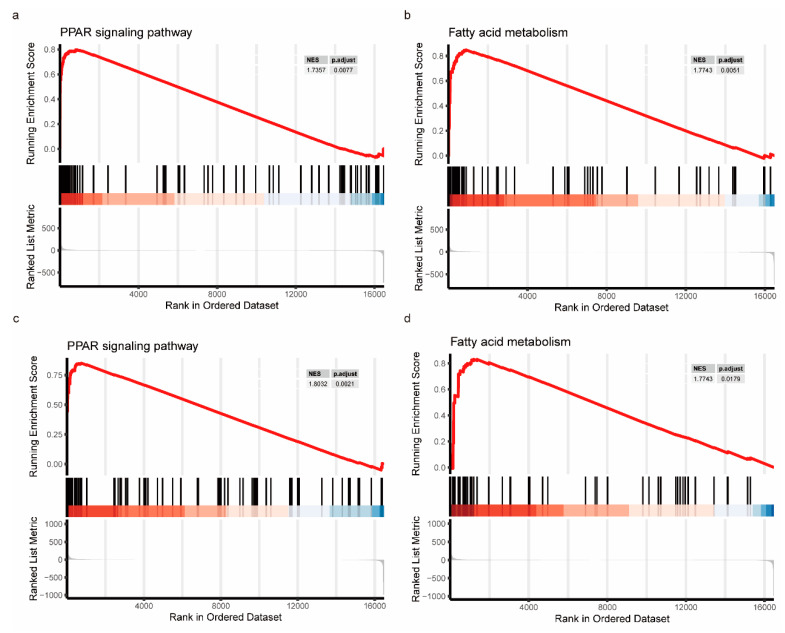
GSEA enrichment analysis of FDX1 in NASH to HCC patients. (**a**) PPAR signaling pathway in NASH patients. (**b**) Fatty acid metabolic signaling pathway in NASH patients. (**c**) PPAR signaling pathway in HCC patients with NASH. (**d**) Fatty acid metabolic signaling pathway in HCC patients with NASH.

We aimed to comprehensively investigate the impact of FDX1 on signaling pathways during the progression from NASH to HCC among patients. We performed separate GSEA analyses for NASH patients and patients with NASH who progressed to HCC. We were surprised to find that the PPAR signaling pathway and fatty acid metabolism were inextricably linked in the NASH patients (Figure 10a,b) and HCC patients with NASH (Figure 10c,d). The specific GSEA results are presented in Supplementary Table S5.

## 4. Discussion

The accumulation of genetic mutations and alterations in cellular signaling pathways due to NASH-induced inflammation and fibrosis may play a key role in the progression of HCC. NASH has been identified as an influential factor in the development of HCC. The utilization of immunotherapy in the treatment of HCC has been validated through clinical trials. However, it has been reported that the therapeutic response to immunotherapy is less efficacious in patients diagnosed with HCC in association with NASH than in individuals with HCC caused by alternative etiologies [23]. Therefore, it is imperative to identify key biomarkers early in the NASH phase, not only to prevent the progression of NASH to HCC but also to optimize the therapeutic response to immunotherapy in patients who have already progressed to HCC via the accumulation of copper ions in the mitochondria, leading to the activation of the tricarboxylic acid cycle and cell death. This process is characterized by the activation of specific signaling pathways and the involvement of certain proteins such as ATP7A and SLC31A1, which regulate copper levels within cells. Cuproptosis is a regulated process, and it can be triggered by various stimuli, such as oxidative stress, DNA damage, and changes in intracellular copper levels. Cuproptosis has been demonstrated to play a role in a variety of diseases and cancers, such as Alzheimer’s disease (AD), Crohn’s disease, and breast cancer (BC) [24]. However, the role of cuproptosis in producing changes in biological function and specific mechanisms in patients with NASH and NASH progressing to HCC is unclear. Therefore, the main purpose of our study was to reveal the relationship between CRGs and NASH patients and patients with HCC and NASH. We also constructed NASH CRCs and HCC CRCs using key CRGs. In NASH and HCC CRCs, we tried to determine the role of immune cell infiltration and identify the molecules that may regulate CRC patterns.

In order to analyze patients with NASH developing into HCC, we used bioinformatics techniques to analyze CRGs. The results indicate that there exist differential CRGs that undergo subtle changes during the progression of NASH to HCC. Specifically, our study found that the expressions of FDX1, LIPT1, and PDHP were significantly elevated in patients with NASH. To date, investigations into the potential association of FDX1, LIPT1, and PDHP with NASH have been lacking. Our study may provide some help in this field. FDX1, DBT, GCSH, and SLC31A1 were significantly downregulated in patients with HCC induced by NASH, while DLAT expression was significantly upregulated. Interestingly, there was a significant difference in FDX1 both in the NASH stage and the NASH to HCC stage. FDX1 facilitates the lipoacylation of DLAT and diminishes iron–sulfur cluster proteins by transforming Cu^2+^ to Cu^+^, thereby triggering cell death through cuproptosis [8]. In addition, FDX1 has the ability to increase ATP production and affect glucose metabolism, amino acid metabolism, and cell proliferation in lung cancer cells [25]. There was a significant positive correlation between FDX1 expression and OS in patients with clear-cell renal-cell carcinoma [26]. The infiltration of neutrophils, Tgd cells, and mast cells is likely to accompany high levels of FDX1 expression [27]. Patients who progress from NASH to HCC are often associated with poor immunotherapy outcomes, and studies have shown that CXCR2 small-molecule inhibitors have the ability to reverse immunotherapy insensitivity in patients [28]. We found that FDX1 was consistently aberrantly expressed in NASH by integrating transcriptomic, single-cell, mouse, and human NASH samples. In light of this, the implementation of early diagnostic strategies and the initiation of appropriate therapeutic interventions for NASH are essential to mitigate the incidence of HCC.

Nomogram models are frequently used to predict outcomes or events based on multiple variables; they can help identify subgroups of patients with different risk levels and potential new treatment options and effectively improve the accuracy and precision of predictions in medical research. Thus, we constructed NASH-related nomograms based on the expression of FDX1, LIPT1, and PDHP. The NASH to HCC nomograms were constructed based on the expression of FDX1, DBT, GCSH, and SLC31A1. After the DCA and CICA evaluation, it was further confirmed that our constructed NOM could be used to evaluate patients with a diagnosis of NASH to HCC, which also provided some clinical insight. The characteristics of the non-negativity constraint of the Nonnegative Matrix Factorization algorithm were utilized to help us explore in depth the biological changes that potentially occur in the NASH CRC and HCC CRC models. The enrichment results revealed that the PPAR signaling pathway, organic acid biosynthesis process, organic acid catabolic process, fatty acid metabolism process, blood microparticles, and oxidoreductase activity are inextricably linked with the key modules of genes extracted using the WGCNA algorithm. Recent literature has demonstrated that there is a disturbance in the metabolism of organic acids and fatty acids in patients with NASH and HCC. Organic acid biosynthesis, organic acid catabolism, and fatty acid metabolism are all biological processes that are involved in the metabolism of these compounds. Blood microparticles, which can be released from cells such as platelets and red blood cells, have been linked to various physiological and pathological processes, including blood clotting, inflammation, and angiogenesis. Oxidoreductase activity, an enzyme activity that catalyzes oxidation–reduction reactions, has also been found to be involved in the pathogenesis of these diseases. The over-accumulation of fat in NASH (non-alcoholic steatohepatitis) leads to the activation of the PPAR signaling pathway, and the over-activation of this signaling pathway also causes liver lesions. In HCC, the activation of the PPARγ signaling pathway can lead to the promotion of cancer cell growth and proliferation, and it can also create a pro-tumorigenic environment. In addition, the PPAR signaling pathway can also promote the anti-tumor immune response and weaken the efficacy of immunotherapy. Our study results are highly consistent with this observation.

Next, we screened five genes (CRP, SRC, TAT, CXCL10, and ACTA1) according to the degree of protein–protein interactions. Investigating alterations in these genes may potentially provide support for enhancing second-line immunotherapy [7]. CRP is a protein produced by the liver that increases when inflammation or infection occurs in the body [29,30]. Patients with NASH have high levels of CRP expression. High levels of CRP may be an alarming factor in the progression of liver disease [31]. SRC promotes lung cancer cell invasion and metastasis through the activation of each TLR4-NF-κB pathway [32]. TAT is associated with the progression of inflammatory bowel disease (IBD). TAT is elevated in the intestine of IBD patients, and its expression is positively correlated with the severity of intestinal inflammation and tissue damage [33]. An excessive accumulation of lipids in the liver can promote M1 macrophage polarization to facilitate their secretion of CXCL10 exacerbating nutritional steatohepatitis [34]. ACTA1 is a potential biological indicator of head and neck squamous cell carcinoma [35].

### Limitations

The limitations of our research are as follows: The first limitation is the limited number of samples in the dataset that we used for the single-cell analysis, GSE210501, which may not adequately represent the heterogeneity of immune cells in NASH. The second problem with this study is that no original sample sequencing was conducted, and the results of the publicly available dataset were not adequately validated. Third, our study lacks a link between the immune microenvironment and second-line immunotherapy. Finally, our animal experiments did not delve into the specific mechanisms of only FDX1′s impact in terms of bioinformatics predictions.

## Data Availability

The article contains the data that were utilized to substantiate the conclusions of this research.

## Supplementary Materials

The following supporting information can be downloaded at: https://www.mdpi.com/article/10.3390/medicina59091639/s1, Table S1: Cuproptosis related gene; Table S2: KEGG analysis results; Table S3: GO analysis results; Table S4: PPI results; Table S5: GSEA results.
